# HEALTHCARE AIDE-FOCUSED INTERVENTIONS TO IMPROVE PAIN MANAGEMENT IN LONG-TERM CARE

**DOI:** 10.1093/geroni/igac059.2035

**Published:** 2022-12-20

**Authors:** Jennifer Knopp-Sihota, Megan Nuspl, Tara MacGregor, Jennifer Reeves, Ahsan Saleem

**Affiliations:** Athabasca University, Edmonton, Alberta, Canada; University of Alberta, Edmonton, Alberta, Canada; University of Alberta, Edmonton, Alberta, Canada; University of Alberta, Edmonton, Alberta, Canada; University of Alberta, Edmonton, Alberta, Canada

## Abstract

Pain is endemic for residents of long-term care homes, with many residents experiencing pain daily. Given that healthcare aides provide most daily care for residents, they are ideally situated to deliver timely assessment and non-drug interventions for managing resident pain. In this Cochrane-style systematic review, we searched 7 databases to identify intervention studies that included long-term care residents aged ≥60 years who received interventions to reduce chronic pain. Interventions were either delivered by healthcare aides at the resident level or were directed at healthcare aides to improve their pain management practices. We screened 400 titles/abstracts and 152 full-text articles. Nine studies met inclusion criteria and were included in a narrative review. Due to the limited number of studies and variety of study designs, data were insufficient to perform meta-analyses or thematic analysis. Three studies described pain interventions delivered by healthcare aides at the resident level reporting significant improvement of pain. Six studies described pain interventions delivered to healthcare aides. Results of these interventions were inconsistent; 2 reported significant improvements in pain-related outcomes (e.g., resident pain, monitoring of pain), 3 reported insignificant changes, and 1 reported a positive correlation between measured pain and pain medication use. We concluded that despite the paucity of research in this area, this systematic review provides preliminary support for pain interventions by healthcare aides for long-term care residents. Future research exploring interventions for healthcare aides to take greater roles in pain management could unlock further improvements in resident care.

